# Deciphering the enigma of neuroendocrine prostate cancer

**DOI:** 10.1172/JCI164611

**Published:** 2022-11-01

**Authors:** Fatima Karzai, Ravi A. Madan

**Affiliations:** Genitourinary Malignancies Branch, National Cancer Institute, Bethesda, Maryland, USA.

## Abstract

Despite the clinical advances in managing metastatic prostate cancer in the last 20 years, treatments for patients with metastatic disease only offer a brief respite from disease progression, especially after first-line therapies. Research into treatment resistance has defined a subset of patients with neuroendocrine differentiation of their prostate adenocarcinoma. Although neuroendocrine findings in conjunction with prostate adenocarcinoma can be seen in pathology samples at all stages of disease, the neuroendocrine variant of prostate cancer associated with poor outcomes occurs in approximately 20% of men with advanced disease. In this issue of *JCI*, Zhao, Sperger, and colleagues present data for a promising biomarker platform that can detect neuroendocrine prostate cancer after serial sampling of patients’ blood with a high degree of sensitivity and specificity. This assay will be tested in several current and future trials to better define its potential clinical role and perhaps provide a greater understanding of neuroendocrine prostate cancer itself.

## Diagnosing neuroendocrine prostate cancer

The last two decades have seen a remarkable expansion of therapeutic options that improve survival for men with metastatic prostate cancer, yet, for most patients, these treatments ultimately lose efficacy over time (1). Many of these therapies are proficient at targeting the androgen receptor, whose signaling axis is a primary driver of cellular proliferation in prostate adenocarcinoma. Substantial investigations have uncovered modifications in the androgen receptor pathway as a common form of treatment resistance in prostate cancer (2). Further studies and clinical experience have identified a neuroendocrine variant of prostate adenocarcinoma that has been associated with worse clinical outcomes (3–5). Studies suggest that about 20% of men with metastatic prostate cancer will develop a form of prostate adenocarcinoma with neuroendocrine features (4). The population of neuroendocrine prostate cancer (NEPC) cells can be characterized with a genomic signature consistent with loss of TP53 and RB1, epigenetic alterations, and diminished dependence on androgen receptor signaling (4, 6).

While research in NEPC is proficient, the clinical entity remains somewhat of a mysterious specter in clinical practice where concerns of a neuroendocrine dedifferentiation abound as patients experience disease progression on initial therapies targeting the androgen receptor. The clinical approach for treating men with advanced prostate cancer requires a broad perspective when considering the possibility of NEPC, a transformation that likely occurs in one of five patients, highlighting that this phenotype is more of an exception than the rule (4). Neuroendocrine findings in prostate cancer patients are sometimes present at diagnosis, but may also arise after androgen deprivation or androgen receptor targeting (7). Speculation exists that NEPC may lurk in the background of patients beyond the 20% expected to develop this potential change in prostate cancer adenocarcinoma lineage. However, without a way to identify these patients it is difficult to justify a change in the clinical approach.

Clinicians often rely on histology findings to diagnose NEPC. While this approach focuses on chromogranin and synaptophysin expression and may imprecisely define NEPC, histological definitions from biopsies are often more available than genomic profiling (3, 7, 8). When we delve into pathological definitions it becomes clear that NEPC as defined on a pathology report in the clinic may mean something slightly different than the approximately 20% of patients with TP53- and RB1-loss and a poor clinical course. Neuroendocrine cells are present in the benign prostate epithelium and thus can be seen with adenocarcinoma cells within the context of a biopsy (7). Thus, prostate cancer with scattered foci of neuroendocrine staining may be expected and frequently described as prostatic adenocarcinoma with neuroendocrine differentiation. Indeed, such findings may be common at initial diagnosis, regardless of Gleason Score, and not associated with poor outcomes relative to patients without such neuroendocrine findings (9). In a clinical setting, neuroendocrine staining is not always requested without clinical concern. This scenario can lead to a degree of ascertainment bias, when apparently aggressive disease is described as adenocarcinoma with neuroendocrine features. Such a finding may be a common variant of adenocarcinoma, either at presentation or with increasing frequency, after treatment with androgen–deprivation based therapies (7). Thus, clinicians and researchers alike need to take great care in describing, defining, and reacting to NEPC as opposed to the more common adenocarcinoma with neuroendocrine features.

A further review of the pathology of NEPC also defines small cell prostate cancer as a rare — perhaps 1%–2% — but virulent and clinically important subset, distinct from adenocarcinoma with neuroendocrine features. Small-cell cancer of the prostate may be seen at diagnosis or after treatment and may exist in parallel with more conventional adenocarcinoma cells. It is often characterized by rapid tumor growth and a disproportionately low–serum PSA (8). Although the literature often uses the descriptors “small cell” and “neuroendocrine” for prostate cancer variants interchangeably or even together, it is clear in the outcome analyses that patients with a more pure-form small-cell prostate cancer have substantially shorter survival — with a median of approximately 12–18 months — than patients with neuroendocrine histologies — approximately 30–36 months — perhaps due to a higher quantity of mixed cells that retain adenocarcinoma features (4, 5). Small-cell cancer of the prostate can be defined in a way that is clearly distinct from adenocarcinoma with neuroendocrine features, as stated in Pirimi et al., “cytoplasm, indistinct cell borders, nuclear molding, fine salt-and-pepper chromatin, lack of prominent nucleoli, extensive tumor necrosis, apoptosis, high mitotic rate, and nuclear fragility” (7).

The distinction between small-cell prostate cancer and adenocarcinoma with neuroendocrine features is critical in the clinic. Despite poor outcomes, small-cell cancer of the prostate is akin to small cell of the lung, and patients can have notable clinical benefit and short-term, substantial responses to a platinum-based regimen more commonly used in small lung cancer. Although adenocarcinoma of the prostate with neuroendocrine differentiation may respond to platinum-based therapies, it is not immediately clear at what point in this lineage transformation standard prostate therapies, such as androgen receptor targeting, need to be abandoned for a small-cell regimen. Prematurely curtailing standard prostate cancer therapies in patients diagnosed with adenocarcinoma with neuroendocrine features in favor of a purely small-cell regimen may lead to increased toxicity and thus diminished survival. Thus, for patients with neuroendocrine findings on a biopsy, it is imperative to understand the morphology from a pathology standpoint and/or establish the underlying genomics. The word “neuroendocrine” contained in a pathology report may not be sufficient as a trigger to abandon standard prostate cancer therapies for a small cell lung cancer regimen.

## Developing a biomarker

A complex clinical scenario such as the one described above is highly predicated on the ability to obtain tissue via biopsies, which, for many reasons, including the fact that prostate cancer is predominantly bone-based, can be difficult. The lack of a readily available and validated biomarker, beyond tissue, further complicates the clinical situation where practitioners may suspect a phenotypic change of the adenocarcinoma to a more neuroendocrine phenotype. It is in this clinical context that we see the potential value for the biomarker strategy presented by Zhao and Sperger et al. in this issue of the *JCI* (10). The authors developed a circulating tumor cell (CTC) multiplex RNA quantitative PCR assay and profiled 116 longitudinal samples from 17 patients (seven of which had NEPC) and also evaluated 265 patients from three trials involving antiandrogen therapies. The CTC assay was successful in detecting NEPC with a sensitivity of 53% and a specificity of 91%, but when serial samples were evaluated from a given patient, the CTC assay was able to detect 100% of the patients with NEPC. Baseline findings of neuroendocrine CTCs were associated with worse clinical outcomes. As the authors indicated, biopsies are limited in their frequency, but the ability to use a CTC assay to evaluate serial blood draws in the clinic would be an important clinical advance, allowing clinicians to identify patients experiencing a lineage transition to a more aggressive neuroendocrine phenotype (10).

## Future opportunities

Zhao and Sperger et al. also report that this assay was able to detect the emergence of neuroendocrine characteristics even while androgen receptor signaling has been maintained (10). This assay creates the intriguing opportunity (with serial CTC assessments) to better define the continuum that exists between prostate adenocarcinoma and NEPC. The authors will further evaluate this CTC assay in multiple current and future clinical trials. With this prospective data, perhaps we can better understand how evolving NEPC can be treated, without abandoning effective antiandrogen, or other standard prostate cancer therapies, prematurely. This assay could also be used to enrich future trials for patients with a neuroendocrine phenotype, thereby providing a better opportunity to investigate emerging treatments for NEPC.

The ability to define and effectively treat the spectrum of NEPC is clearly an unmet need in the clinic. Furthermore, more research into the most virulent neuroendocrine subset, small-cell prostate cancer, is required to better understand what therapies, in addition to platinum-based lung cancer regimens, could be effective. The assay and supporting data presented by Zhao and Sperger et al. (10) create optimism that the shroud of mystery cloaking NEPC will soon be lifted, and better biological understanding will bring about improved clinical management and outcomes for prostate cancer patients.

## Version 1 11/01/2022

Electronic publication
